# Effects of MeJA on *Arabidopsis* metabolome under endogenous JA deficiency

**DOI:** 10.1038/srep37674

**Published:** 2016-11-24

**Authors:** Jingjing Cao, Mengya Li, Jian Chen, Pei Liu, Zhen Li

**Affiliations:** 1 State Key Laboratory of Plant Physiology and Biochemistry, College of Biological Sciences, China Agricultural University, Beijing, 100193, China; 2 College of Resources and Environmental Sciences, China Agricultural University, Beijing, 100193, China

## Abstract

Jasmonates (JAs) play important roles in plant growth, development and defense. Comprehensive metabolomics profiling of plants under JA treatment provides insights into the interaction and regulation network of plant hormones. Here we applied high resolution mass spectrometry based metabolomics approach on *Arabidopsis* wild type and JA synthesis deficiency mutant *opr3*. The effects of exogenous MeJA treatment on the metabolites of *opr3* were investigated. More than 10000 ion signals were detected and more than 2000 signals showed significant variation in different genotypes and treatment groups. Multivariate statistic analyses (PCA and PLS-DA) were performed and a differential compound library containing 174 metabolites with high resolution precursor ion-product ions pairs was obtained. Classification and pathway analysis of 109 identified compounds in this library showed that glucosinolates and tryptophan metabolism, amino acids and small peptides metabolism, lipid metabolism, especially fatty acyls metabolism, were impacted by endogenous JA deficiency and exogenous MeJA treatment. These results were further verified by quantitative reverse transcription PCR (RT-qPCR) analysis of 21 related genes involved in the metabolism of glucosinolates, tryptophan and α-linolenic acid pathways. The results would greatly enhance our understanding of the biological functions of JA.

Metabolomics utilizes high-throughput methods, such as high resolution mass spectrometry and nuclear magnetic resonance to obtain comprehensive information of metabolites in a given biological system^1^. UPLC-HRMS has been regarded as one of the most promising tools for metabolic profiling with improved accuracy and resolution^2^,^3^,^4^. Metabolomics have been widely used in biology study, especially in the field of biomedical research^5^,^6^,^7^. Plant metabolomics combined with other biology approaches were used to investigate gene function and biological processes. Large scale metabolomics profiling of plant materials can identify compounds involved in key metabolic pathways for plant growth and stress response, reveal correlation/interaction between metabolites from distinct metabolic pathways and construct a metabolite regulatory network. Chen *et al*. investigated the effects of drought stress on rice metabolome using MS2T based approach and found possible regulation coordination of abscisic acid (ABA) with serotonin derivatives and polyamine conjugates^8^. This study also found some C-glycosylated flavones as potential biomarkers for discrimination between two rice subspecies. Metabolomics was also used to illustrate the contrasting effects of metabolites on herbivore resistance in leaves and roots of maize upon attack by herbivores^9^.

Jasmonate acid (JA) is an important phytohormone, it regulates a wide variety of developmental processes and mediates the response to various environmental stresses in higher plants. JA plays essential roles in seed germination, flowering and fruit development, leaf abscission and senescence^10^,^11^. JA also regulates plant stress response such as insects, fungal pathogens, UV radiation, ozone and other biotic or abiotic stresses^12^,^13^,^14^,^15^,^16^,^17^. The biosynthesis of JA starts with the conversion of linolenate to 12-oxo-phytodienoate (OPDA) in the chloroplast. OPDA is then transported into peroxisome where it is reduced by OPDA reductase^18^ through three cycles of β-oxidation^19^ to produce JA. JA can be reversibly esterified by JMT (JA-methyl transferase) to MeJA^20^ and can be conjugated to amino acids by JAR1^21^ in cytoplasm. Exogenous stimuli induce rapid accumulation of JA in plants and subsequently induce the expression of a number of JA regulated genes.

Mutants in JA synthesis and signaling pathways were used to study the mechanism of JA synthesis as well as the roles of JA in plants^22^,^23^,^24^. These studies focused on phenotypic and molecular features, while at the metabolites level, only a few metabolites such as OPC:8, OPDA, JA, MeJA and JA-Ile^25^,^26^,^27^ were quantified. This is far from enough, as plant metabolites are end products of genes regulation that reflect the real physiological condition within the plant. It has already been demonstrated that plant secondary metabolites, especially plant hormones have an intricate interaction network. Environmental stresses or the application of exogenous hormones can trigger a cascade of responses of different types of plant hormones^28^,^29^,^30^. Comprehensive profiling of plant metabolites of various mutants or under different physiological/stress conditions can provide a perspective view of the plant and provide insights into the interaction and regulation network of plant metabolites.

Most mutants defective in JA synthesis, reception or signaling, such as *aos, dad1, opr3, coi1, myb21* and *myb21 myb24*, are male-sterile due to defects in late stamen development^22^,^23^,^24^,^31^. This phenotype can be rescued efficiently by exogenous MeJA application in JA-deficit mutant *opr3*. The *opr3* mutant, defective in *OPR3* expression, shows dwarf and male-sterile phenotype with insufficient anther filaments elongation, unseasonal anther locule dehiscence and inefficient pollen maturation^32^. *OPR3* encodes OPDA Reductase 3 (OPR3), the key enzyme catalyzing the conversion of OPDA to OPC-8:0 in JA biosynthesis. There are other OPR enzymes in *Arabidopsis* (OPR1 and OPR2) but the efficiency is not up to the level of OPR3^26^. OPR3 co-localizes with the enzymes of β-oxidation in peroxisomes, which catalyze the final steps in the formation of JA^33^. In *opr3* mutant, *OPR3* transcript and JA synthesis were absent in both reproductive and vegetative tissues, but resistance to fungal infection and insect attack retained. Wound induced genes previously known to be JA-dependent were still induced in *opr3* plants, thus, other octadecanoid-derived molecular(s) may be effective for defense in plants in the absence of JA^27^. Although the core signaling transduction pathway and functions of JA are clarified, unsolved questions remain. How does exogenous MeJA restore the function of endogenous JA? Whether endogenous JA can be replaced by exogenous MeJA? How does JA regulate plant metabolites and crosstalk with other plant hormones? Comprehensive profiling of metabolites involved in JA functions and the establishment of plant hormones interaction network is essential for a better understanding of plant defense mechanism.

In the present study, we performed high throughput metabolic profiling to investigate the effects of JAs on *Arabidopsis*. The metabolomes of *Arabidopsis* wild type and *opr3* were compared to study the effect of endogenous JA deficiency due to *OPR3* mutation. In addition, *opr3* plants treated with MeJA at various time points were used to study the effects of exogenous MeJA on plants. The knowledge about differential metabolites identified in wild type, *opr3* and MeJA-treated *opr3* plants helps us understand the functions of JA integrally and dynamically. We obtained a list of hundreds of non-redundant metabolites that exhibited significant variations. The biological functions and metabolic pathways of 109 annotated metabolites were examined. The expressions of 21 genes involved in the annotated metabolic pathways were validated by RT-qPCR. The results would greatly enhance our understanding of the biological functions of JA.

## Results

### Optimization of the extraction methods

Plant metabolites have diverse chemical and physical properties. In order to achieve maximum recovery of the metabolites, the sample extraction procedure needs to be optimized. The extract solvents, the ratio between sample and extract solvent, the duration and temperature of extraction all need to be considered^34^. Aqueous (water) and organic (MeOH/ACN) solvents were used in sequential at various sample/solution ratios to extract metabolites of different polarity (Supplementary Table S1).

With all extraction methods, a strong peak corresponding to polar compounds can be observed as the flow-through peak in the TIC (total ion chromatogram). When the sample to extract solvent ratio was 1:3 (W/V), that peak was the most intensive peak, while peaks corresponding to non-polar and mid-polar compounds showed very weak signal. A small improvement in TIC was observed after increasing the volume of extract solvent. The signal increased significantly after concentrating the extract solution by vacuum concentrator, while there exhibited no obvious difference between ultrasound and heating for extraction (Supplementary Fig. S1).

The numbers of detected peaks (intensity threshold, 5 × 10^6^) were compared between different extraction methods. The peak number increased significantly after changing the ratio of sample to solvent from 1:3 to 1:15 (Supplementary Fig. S2, p = 4.10 × 10^−7^). Similarly, significant increase in peak number was observed after concentrating the sample by vacuum concentrator (p = 5.57 × 10^−6^). There was no significant change in the number of detected peaks between ultrasound and heating (p = 0.273). But, extraction method C had more peaks with higher intensities than extraction method D (Supplementary Fig. S2, insert, p = 0.00254). So, solvent extraction combined with ultrasound and vacuum concentration detected the highest number of peaks and was used for subsequent sample extraction. As shown in Supplementary Fig. S1, no contamination was introduced by this method.

### Data preprocessing and statistical analysis

Extracts from *Arabidopsis* wild type, *opr3* and *opr3* plants treated with MeJA at various time points were analyzed with UPLC-HRMS using a C18 column in the positive ion mode. TIC from different genotypes and treatments were very similar visually, with a few exceptions at retention time 4.51 and 8.23 min (Supplementary Fig. S3). Raw MS data were analyzed with SIEVE software to extract mass spectrometric features, 12016 ion signals were detected (above the 5 × 10^5^ peak intensity threshold). Most of the detected peaks (77.8%) have a coefficient of variation (CVs) equal or less than 20% in 10 replicates (Supplementary Fig. S4).

**Figure 1:**
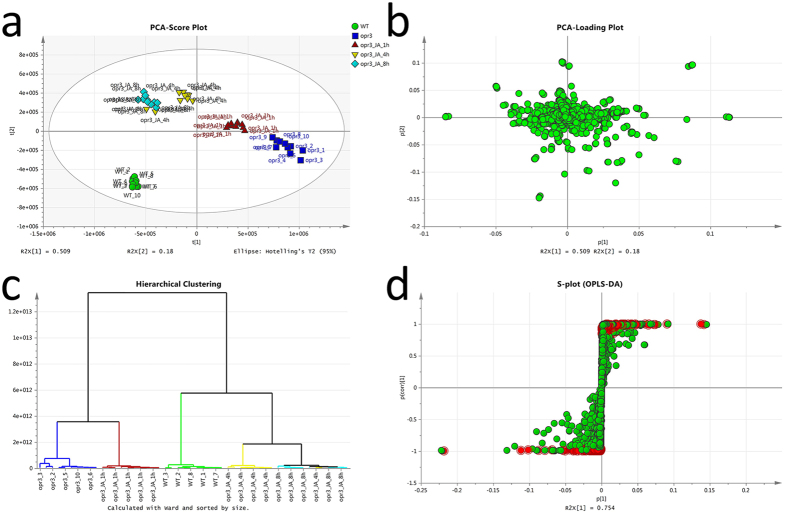
Statistical analysis of normalized dataset. (**a**) Score Plot, (**b**) Loading Plot of Principal component analysis (PCA) and (**c**) Hierarchical cluster analysis (HCA) using all of the 12016 ion signals. (**d**) S-plot of orthogonal partial least squares discriminant analysis (OPLS-DA) of wild type and *opr3* leaf extracts. Compounds with a *p* value less than 0.05 and fold change higher than 2 are highlighted in red.

Principal component analysis (PCA) and hierarchical cluster analysis (HCA) of the 12016 ion signals were performed to reveal intrinsic difference within the signals (Fig. 1a,b and c). Both statistical methods revealed similar relationship between different genotype and treatment groups. PCA score plot showed that the two genotypes were mainly separated along PC1 (50.9%), while PC2 (18.0%) represented difference between *opr3* after MeJA treatments. The long distance along PC1 between wild type and *opr3* plants indicated there are major differences in the metabolome between these two genotypes. The *opr3* migrated gradually along both PC1 and PC2 in the PCA score plot after MeJA treatment. After 8 h, *opr3* moved from the forth quadrant to the second quadrant on the score plot, indicating MeJA treatment can greatly alter the composition of *opr3* metabolome. The relative position between wild type and MeJA treated *opr3* (8 h after treatment) indicated exogenous MeJA can bring some features back to a level comparable to the wild type, while at the same time, the content of other features changed in the opposite direction to that of wild type. HCA revealed that the samples were separated into two major clusters (Fig. 1c). The first cluster included *opr3, opr3* treated with MeJA for 1 h, the second cluster included wild type and *opr3* treated with MeJA for 4 and 8 h. Such phenomenon implied that exogenous application of MeJA may rescue at least part of the features disturbed by *OPR3* deficiency in a time dependent manner.

Orthogonal partial least squares discriminant analysis (OPLS-DA) was carried out to highlight differential features between genotypes wild type and *opr3* (Fig. 1d). With filter conditions: CV < 20%, *p* value < 0.05 and fold change >2, we obtained a matrix containing 754 non-redundant differential mass spectrometric features as putative biomarkers, which were highlighted in red in the S-plot. Features affected by MeJA treatment in *opr3* mutant were screened similarly and 1475 differential features were obtained.

### Identification, classification and pathway analysis of differential compounds

JA regulates plant growth, development and reproduction as well as stress responses. Thus, a wide range of metabolites can be affected by *OPR3* deficiency or MeJA treatment in *opr3* plants. Further analysis was then focused on metabolites that showed significant variations between different genotype and treatment groups (p < 0.05 and fold change >2). Metabolite identification was performed with the help of accurate mass measurement of molecular ions and fragment ions with high resolution. Accurate mass, isotopic pattern and MS/MS spectra were searched against metabolite databases and theoretical fragmentations. It’s impossible to realize thorough and comprehensive metabolite identification due to the diversity of plant metabolites and the incompleteness of metabolite databases. Thus, we established a differential compound library containing 174 compounds with high signal intensity and signal to noise ratio (Supplementary Information Spreadsheet 1). The spreadsheet contains *m/z*, peak intensity, mass error, isotope similarity, MS/MS fragment ions and method used for metabolite identification. These compounds were affected either by the absence of endogenous JA or the application of exogenous MeJA. Accurate and high resolution precursor-product ions pairs combined with reproducible retention time in this library will help identification of the metabolites in the future.

**Figure 2:**
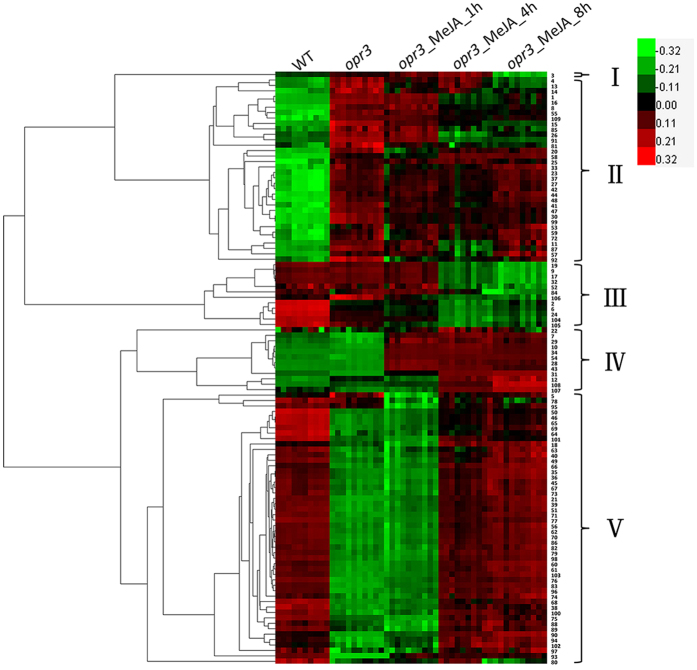
Heat map analysis of the identified compound revealed 5 clusters according to their variation trends. The sequence numbers were corresponding to Supplementary Table S2.

Within the 174 differential metabolites, 109 compounds were identified (Supplementary Table S2). Metabolite identifications can be classified as level II according to MSI criteria based upon their spectral similarities with public/commercial spectral libraries^35^. These compounds included aliphatic and aromatic compounds, amino acids and peptides, carbohydrates and carbohydrate conjugates, organic acids and derivatives and lipids. Heat map analysis of the differential metabolites revealed 5 clusters (Fig. 2). The sequence numbers in Fig. 2 were corresponding to Supplementary Table S2. Cluster II contained almost all of the amino acids and small peptides. Several isothiocyanates, lipids and other compounds were also found in this cluster. These metabolites have higher abundances in *opr3* than in wild type, MeJA treatment down-regulated some of the metabolites. Cluster V contained mainly lipids with a few other compounds. The abundances of these metabolites declined dramatically with endogenous JA deficiency, which can be rescued by exogenous MeJA treatment. Metabolites in these two clusters had opposite variation trends and may be the basis of remedy of endogenous JA deficiency by exogenous MeJA. Cluster III contained lipids and isothiocyanates while cluster IV contained aromatic compounds, glucosinolates and lipids. Metabolites in these two clusters had comparable abundances in wild type and *opr3* plants, but dramatic changes were induced by the application of exogenous MeJA, resulting in either down-regulation (cluster III) or up-regulation (cluster IV) of the metabolites. So, these metabolites are regulated by exogenous MeJA application but not by endogenous JA deficiency. In summary, the functions of endogenous JA can be compensated partially, but not completely by exogenous MeJA at the metabolic level in a time-dependent manner, and endogenous JA cannot be completely replaced by exogenous MeJA.

**Figure 3:**
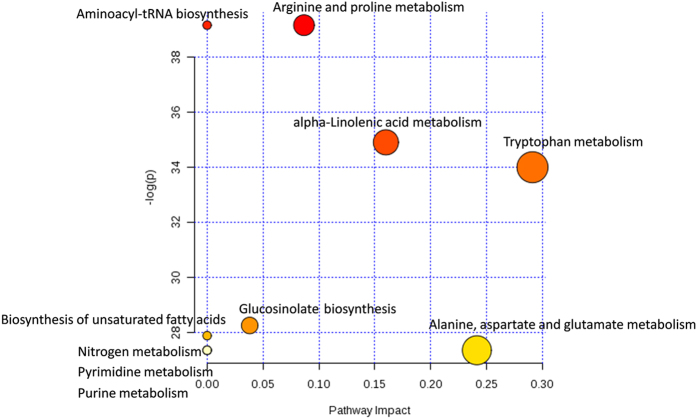
Result of pathway analysis. The *p* values were calculated from the enrichment analysis while the pathway impact values were calculated from pathway topology analysis.

Pathway analysis was performed using MetaboAnalyst 3.0, which uses high-quality KEGG metabolic pathways as the backend knowledgebase^36^,^37^. Peak intensities of the identified compounds were normalized by sum, log transformed, Pareto scaled and factored into the pathway analysis (Supplementary Fig. S5). Ten most relevant metabolic pathways were identified by pathway enrichment and pathway topology analysis (Fig. 3 and Supplementary Table S3). Identified pathways include: arginine and proline metabolism, aminoacyl-tRNA biosynthesis, alpha-linolenic acid metabolism, tryptophan metabolism, glucosinolate biosynthesis, biosynthesis of unsaturated fatty acids, alanine, aspartate and glutamate metabolism, purine metabolism, pyrimidine metabolism and nitrogen metabolism.

### Glucosinolates and tryptophan metabolism affected by *OPR3* deficiency and MeJA treatment

**Figure 4:**
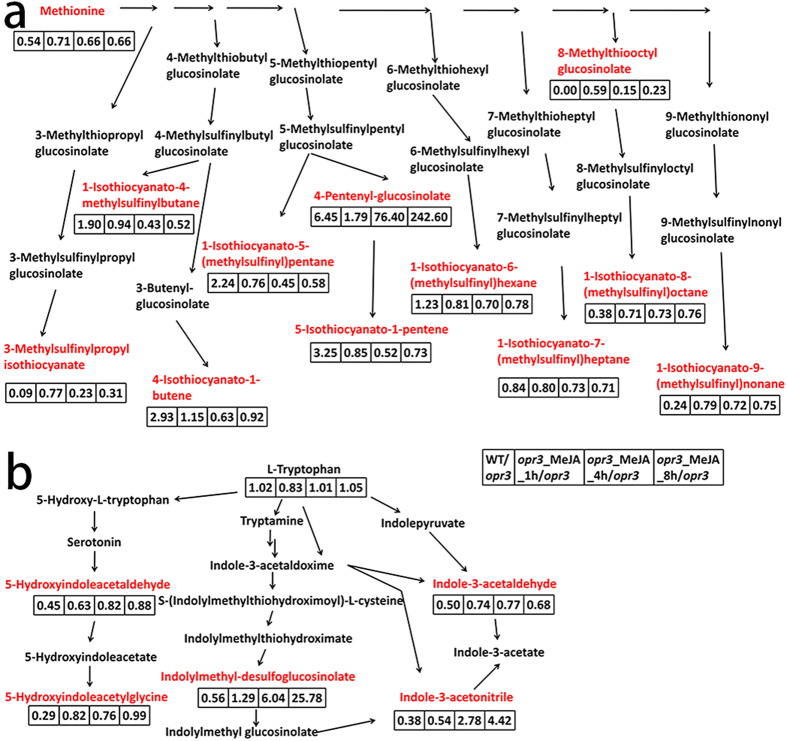
Glucosinolates and tryptophan metabolism affected by *OPR3* deficiency and MeJA treatment. (**a**) Methionine derived glucosinolates metabolism, (**b**) tryptophan metabolism were influenced by *OPR3* deficiency and MeJA treatment. Differential metabolites detected are labeled in red, fold changes compared to *opr3* were displayed in the boxes.

Glucosinolates are important secondary metabolites mainly found in the Brassicaceae family. There are about 40 glucosinolates identified in *Arabidopsis*, mainly derived from methionine and tryptophan^38^. Glucosinolates can be hydrolyzed into isothiocyanates in the presence of myrosinase in response to abiotic stresses. Isothiocyanates are important for both plant defense and human nutrition^39^. We found several methionine derived aliphatic glucosinolates and related metabolites. Among them, 5 exhibited decreased levels in *opr3* and no restoration was observed after MeJA treatment, 5 showed increased levels in *opr3* and slightly declined after MeJA treatment (Fig. 4a).

1-Isothiocyanato-4-methylsulfinylbutane, 4-isothiocyanato-1-butene, 1-isothiocyanato-5-(methylsulfinyl) pentane, 5-isothiocyanato-1-pentene and 1-isothiocyanato-6-(methylsulfinyl) hexane have significantly lower abundances in *opr3*. MeJA treatment resulted in further decrease of these compounds during the first 4 h of treatment and slight recovery was observed after 8 h (Fig. 4a). 1-Isothiocyanato-4-methylsulfinylbutane, a natural product derived from aliphatic glucosinolates, reduces damage by herbivores and inhibits growth of non-host pseudomonas bacteria in *Arabidopsis*^40^. 4-isothiocyanato-1-butene and 5-isothiocyanato-1-pentene display potent antibacterial activity against *A. hydrophila*^41^. 1-isothiocyanato-5-(methylsulfinyl)pentane is a 1-isothiocyanato-4-methylsulfinylbutane analog which exhibits potent anti-inflammatory properties^42^. The variation of these metabolites implied that the antibacterial activity and insect resistant of plant might be impaired in the *opr3* mutant which can’t be mitigated by exogenous MeJA treatment.

Metabolite variation patterns similar to methionine (increased levels in *opr3* and slightly declined after MeJA treatment, Fig. 4a) were observed with 3-methylsulfinylpropyl isothiocyanate, 1-isothiocyanato-7-(methylsulfinyl)heptanes, 8-Methylthiooctyl glucosinolate, 1-isothiocyanato-8-(methylsulfinyl)octane and 1-isothiocyanato-9-(methylsulfinyl)nonane (Fig. 4a). Guo *et al*. found that 1-isothiocyanato-8-(methylsulfinyl)octane could inhibit germination of lettuce seeds and affects growth of roots of lettuce seedlings^43^. The increased levels of these isothiocyanates in *opr3* implied that plant growth may be inhibited in response to the deficiency of endogenous JA. This is consistent with the dwarf phenotype of *opr3*. Endogenous JA deficiency-induced isothiocyanates accumulations were slightly relieved by MeJA treatment in a time dependent manner. This must be associated with the function recovery in *opr3* by exogenous MeJA. Almost all aliphatic isothiocyanates detected showed no significant difference after MeJA treatment (Fig. 4a). Ku *et al*. found that exogenous MeJA treatment increases glucosinolate biosynthesis in Kale leaf tissue^44^. So, the hydrolysis of glucosinolates may be not significantly influenced by exogenous MeJA in 8 h in *opr3*.

The lack of endogenous JA can reduce the content of certain isothiocyanates, while at the same time; it can increase the content of other isothiocyanates. The application of exogenous MeJA, on the other hand, generally resulted in slightly decreased isothiocyanates content. This phenomenon indicated that some adverse impacts from endogenous JA deficiency may be alleviated by exogenous MeJA application. In addition to antibacterial activity and insect resistance, isothiocyanates also act as heat tolerance enhancers in plant^45^. Khokon *et al*. found that exogenous allyl isothiocyanates (AITCs) induce stomatal closure accompanied and mediated by the production of ROS and NO^46^. This process requires MeJA priming and AITC signaling shares some components with JA signaling. In conclusion, there are close associations between glucosinolates metabolism and JA in plant growth and stress response.

Tryptophan metabolism was also impacted by endogenous JA deficiency and exogenous MeJA treatment. Tryptophan is the metabolic precursor of many important secondary metabolites. Serotonin and indoleacetic acid (IAA) are two major secondary metabolites derived from tryptophan metabolism pathway. They are essential for plant growth and development, plant morphogenesis, pathogen defense, plant-insect interactions and other stress responses^47^,^48^. The content of tryptophan remained relatively stable between different genotype and treatment groups. 5-Hydroxyindoleacetaldehyde and 5-Hydroxyindoleacetylglycine, metabolites of serotonin, showed higher abundances in *opr3* plants (Fig. 4b). The synthesis and metabolism of serotonin may be activated by endogenous JA deficiency. MeJA treatment resulted in reduction of them during the first 4 h. Their concentrations rose again to the levels of untreated mutant after 8 h. Such phenomenon indicated MeJA has antagonistic effect on serotonin metabolism. Tryptophan-dependent IAA synthesis contains four different pathways and we found three differential metabolites in them. For indolylmethyl-desulfoglucosinolate and 3-indoleacetonitrile, the variation between wide type and *opr3* samples was small. Exogenous application of MeJA resulted in dramatic increase of these two metabolites after 4 h of treatment, consistent with the fact that these two metabolites are at the downstream of the metabolism pathway, a series of reactions are involved which may result in slow response of the metabolite. For indoleacetaldehyde, higher intensity was found in *opr3* plants, and MeJA treatment resulted in decreased intensities. This variation pattern was similar to methionine and related glucosinolates. The opposite variation trends between indoleacetaldehyde and indoleacetonitrile after MeJA treatment under nearly constant level of tryptophan indicated that plants have redundant pathways for IAA synthesis and the application of MeJA can affect the synthesis of IAA by different pathways. It was found that IAA decreased significantly while serotonin increased significantly under various stress conditions in rice^49^. Our finding showed constant tryptophan content between different genotype and treatment groups and antagonistic variation between different tryptophan metabolism pathways after MeJA treatments in *opr3* plants. Taken together, we suggest a negative correlation between tryptophan derived IAA and serotonin synthesis. The activation of serotonin synthesis in *opr3* may result in inhibition of IAA synthesis, which is consistent with the dwarf phenotype of *opr3* mutant. Serotonin involves in almost all aspects of plant growth, development and stress defense^50^. The activation of serotonin synthesis and metabolism may be used to help regulate plant growth and stress response to make up the lack of endogenous JA in *opr3* plants.

### Lipid metabolism affected by *OPR3* deficiency and MeJA treatment

Lipid metabolism, especially fatty acyls metabolism, was impacted extensively by endogenous JA deficiency and exogenous MeJA treatment. We found 45 lipids that covered five lipid subcategories based on lipid classification system of LIPID MAPS, including, fatty acyls (23), prenol lipids (13), sterol lipids (7), sphingolipids (1) and glycerophospholipids (1) (Supplementary Information Spreadsheet 1).

**Figure 5:**
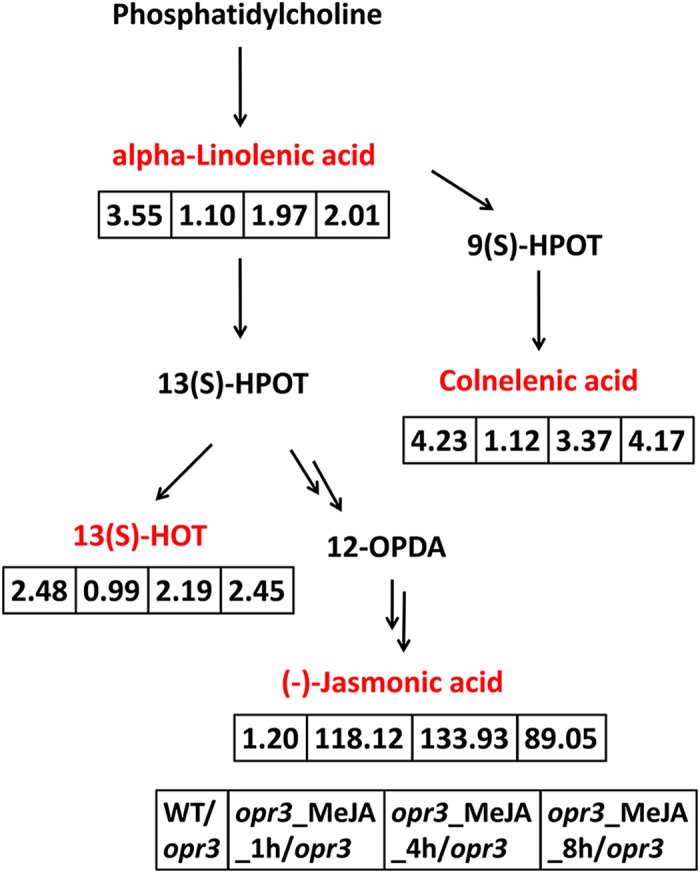
alpha-linolenic acid metabolism was influenced by *OPR3* deficiency and MeJA treatment. Differential metabolites detected are labeled in red, fold changes compared to *opr3* were displayed in the boxes.

In plants, fatty acyles and their derivatives are recognized as signaling molecules to control various biological processes including, cell membrane construction, intracellular signal transduction, protein modification^51^. They can significantly impact gene expression and metabolism in plant-microbe and plant-herbivore interactions^52^. α-linolenic acid, colnelenic acid and 13(S)-HOT, three key components of α-linolenic acid metabolism, showed decreased levels in *opr3* and increased levels after MeJA treatment. Linolenic acid, a stress signal by itself 53, is one of the major unsaturated fatty acids in plants. Under stress conditions, it can be released into the plastid. This is the initial step of unsaturated fatty acid metabolism. Catalyzed by lipoxygenase, linolenic acid can produce a series of bioactive compounds, such as JA, MeJA, and 12-OPDA. The higher abundances of linolenic acid and their metabolites (except JA) in wild type indicated that plant keep a certain amount of “raw material” in reserve for the synthesis of JA when needed. In *opr3*, such reserve was greatly reduced (75% reduction in signal intensities), application of exogenous MeJA can alleviate the situation in a time dependent manner. The content of colnelenic acid and 13(S)-HOT can reach levels of the wild type after applying MeJA for 8 h, indicating exogenous MeJA can trigger its synthesis pathway by itself, but the lack of OPDA reductase in *opr3* routed linolenic acid metabolism to pathways other than JA synthesis, as indicated by the high intensities of 13(S)-HOT and colnelenic acid in MeJA treated *opr3* (Fig. 5). This finding suggested that JA is regulated in plant in a close-loop circuit. It has been reported that overexpression of fatty acid desaturase increased level of linolenic acid, accompanied by increase of drought resistance, salt^54^ and chill tolerance^55^. These findings suggested *opr3* have weaker resistance towards abiotic stress due to decreased content of linolenic acid. But, such deficiency can be made up by exogenous MeJA application. This is consistent with the result of Vijayan *et al*., exogenous MeJA can protect mutants deficient in JA synthesis from pathogen infection and strengthen disease resistance of plants^56^. In addition to the fatty acyles mentioned above, we have found other fatty acyles negatively regulated by *OPR3* deficiency and positively regulated by MeJA treatment (Supplementary Fig. S6). Their variation patterns were similar to that of α-linolenic acid.

JA is also involved in the metabolism of several prenol lipids and sterol lipids (Supplementary Fig. S7). Two types of variation pattern were observed. For 4, 5-Dihydrovomifoliol, 3-Methyl-alpha-ionyl acetate, 5-Hydroxy-p-mentha-6, 8-dien-2-one and (1beta, 2beta, 5beta)-p-Menth-3-ene-1,2,5-triol, comparable intensities were observed in wild type, *opr3* and *opr3* treated with MeJA for 1 h. *opr3* plants treated with MeJA for 4 and 8 h showed significantly reduction of these compounds (Supplementary Fig. S7). Other prenol and sterol lipids showed variation patterns similar to that of α-linolenic acid.

### Amino acids and small peptides

As shown in Figs 2 and 3 and Supplementary Information Spreadsheet 1, there are other metabolites and pathways influenced by endogenous and exogenous JA. Amino acids and small peptides are among them (Supplementary Fig. S8). L-glutamine and proline, two essential amino acids, showed increased content in *opr3*. After MeJA treatment, these two amino acids showed slightly different trends of variation. The content of proline increased initially and then dropped suddenly after 8 h. While for L-glutamine, the decrease occurred at 4 h after treatment. Glutamine involves in plant ammonia assimilation and provides nitrogen for amino acid synthesis. The increased level of glutamine in *opr3* indicates enhanced nitrogen uptake of plants in the absence of JA. This situation is alleviated by exogenous MeJA. Proline metabolism has roles in cellular redox buffering, energy transfer, programmed cell death, osmotic balance regulation and plant pathogen interactions^57^. The increased level of proline in *opr3* indicates an increased level of resistance in the mutant, and application of MeJA further enhanced such situation until 8 h after treatment when the concentration of JA in plant started to decrease (Fig. 5). These findings suggest there are associations between proline and JA in plant growth and stress response.

It is noteworthy that many dipeptides, tripeptides and amino acid metabolites were induced by *OPR3* deficiency, they showed higher intensities in *opr3* than in the wild type. This situation remained after exogenous MeJA treatment (Supplementary Fig. S8). Protein contents were measured by Bradford assay and no obvious difference was observed between WT and *opr3* (Supplementary Fig. S9). Thus, the increased content of small peptide was not induced by large scale degradation of proteins in *opr3*. Small peptides may act as bioactive or signaling molecules. They have antioxidant, antihypertensive, anticarcinogenic, antimicrobial, and immunomodulatory activities in animals^58^. Here, we found many small peptides (especially dipeptides) that showed significant variation in the absence of endogenous JA, these small peptides may act as signaling molecules in plants and collaborate with JA in plants growth and stress regulation. Further study is needed to elucidate the role of small peptides in plant stress defense.

### Quantitative analysis of expression of glucosinolates, tryptophan and α-linolenic acid metabolism related genes

To further verify our observation from metabolomics experiments, we examined the expression of 21 genes in MeJA-treated leaves of *opr3*, untreated leaves of *opr3* and wild type *Arabidopsis* by RT-qPCR. These genes were selected based on their functions in the specific metabolic pathways identified in this study (glucosinolates, tryptophan and α-linolenic acid).

**Figure 6:**
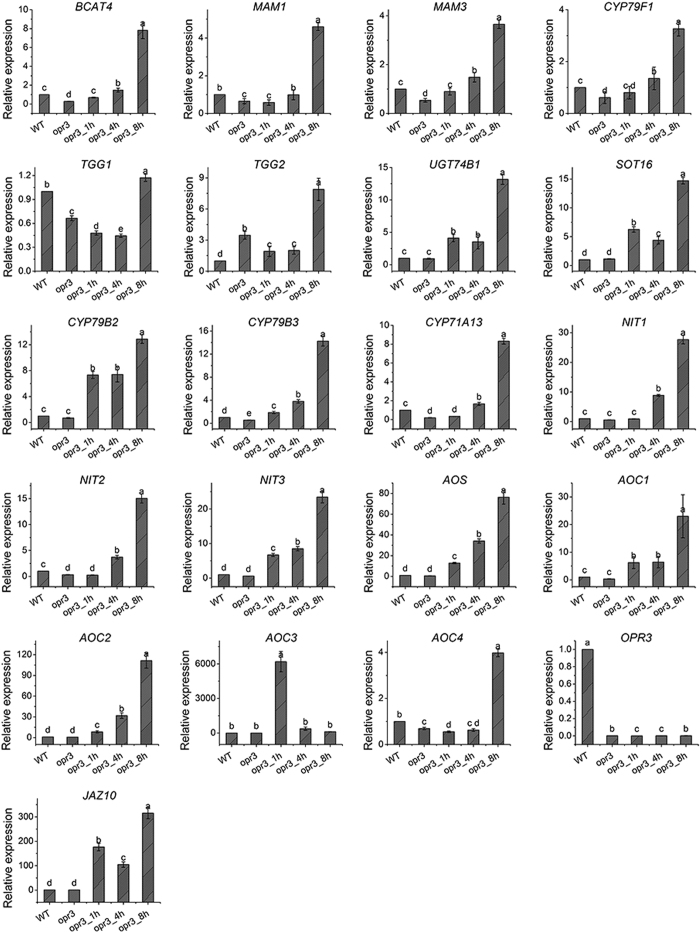
Gene expression analysis by RT-qPCR. 21 genes were selected based on their functions in specific metabolic pathways (glucosinolates, tryptophan and α-linolenic acid). Data represent mean ± SD from three biological and three technical replicates. Superscript letter indicates the result of ANOVA test (p < 0.05).

*BCAT4*, encoding branched-chain aminotransferase 4, catalyzes the initial step of aliphatic glucosinolate biosynthesis from methionine. The chain-elongated methionines are converted by MAM1 and MAM3 and further metabolized by CYP79F1 to produce aldoximes. *TGG1* and *TGG2* specifically expressed in aerial part of the plants and encode myrosinase, which catalyses the hydrolysis of glucosinolates to form isothiocyanates. The expression levels of these genes (except *TGG2*) were lower in *opr3* than in wild type and this deficiency can be relieved by MeJA treatment in a time dependent manner (Fig. 6). Thus, RT-qPCR results confirmed that the biosynthesis and hydrolysis of aliphatic glucosinolates were negatively regulated by endogenous JA deficiency and positively regulated by exogenous MeJA treatment. This indicated that plant resistance to bacteria and insects may be impaired in the absence of endogenous JA and this impairment may be rescued by exogenous MeJA. Notably, almost all aliphatic isothiocyanates detected showed slow decrease in content after MeJA treatment according to our metabolomics data, while the expression of *TGG1* and *TGG2* decreased initially after MeJA treatment in *opr3* then increased after 8 h of treatment. Taken together, we can conclude that for the hydrolysis of aliphatic glucosinolates, the change of gene expression may has not been passed to the metabolites level even after 8 h of MeJA treatment.

*UGT74B1* (encodes a UDP-glucosyl transferase) and *SOT16* (encodes a desulfo-glucosinolate sulfotransferase) are critical genes involved in indolic glucosinolate biosynthesis from tryptophan. There were no significant differences in their expressions between wild type and *opr3*. In contrast, MeJA treatment induced significant increases of their expressions in *opr3* (Fig. 6). Similar variation trend was observed for their metabolic intermediate indolylmethyl-desulfoglucosinolate in this pathway (Fig. 4b). For tryptophan-dependent auxin biosynthesis, indole-3-aldoxime is converted by CYP79B2 and CYP79B3 and further catalyzed by CYP71A13 to produce indole-3-acetonitrile. Subsequently, three nitrilases (NIT1, NIT2 and NIT3) catalyse the terminal activation step in IAA biosynthesis. The lack of endogenous JA resulted in slight down-regulation of the expression of these genes, while application of exogenous MeJA resulted in significant up-regulation of these genes, suggesting JA has a positive correlation with tryptophan-dependent auxin biosynthesis (Fig. 6).

*AOS (allene oxide synthase*)*, AOCs (allene oxide cyclase1, 2, 3 and 4*) and *OPR3* are key genes involved in the synthesis of JA. The upstream genes, *AOS* and *AOCs*, showed comparable expression levels in wild type and *opr3*. MeJA treatment resulted in significant increases of these genes in *opr3* (Fig. 6). *OPR3* was not expressed in *opr3* in any conditions as expected. The downstream gene involved in JA biosynthesis/signaling included *JAZ10 (jasmonate-zim-domain protein 10*), which regulates JA signaling pathway. The expression of this gene showed extremely low levels in wild type and non-treated *opr3*. The transcript level of *JAZ10* increased significantly and quickly after MeJA treatment, indicating *JAZ10* is actively involved in response to JA stimulus.

Most of these genes showed lower level of expression in *opr3*, indicating endogenous JA is required for their proper expression. Most of the genes also showed late response upon MeJA treatment in *opr3*. The highest level of expression was observed at 8 h after MeJA treatment, indicating a series of action is needed to initiate expression of these genes.

## Discussion

High resolution mass spectrometry based metabolomics approach was used as a discovery tool to identify metabolites actively involved in JA synthesis and signaling in *Arabidopsis*. Multivariate statistic analyses revealed that there is significant difference between the metabolomes of *Arabidopsis* wild type and JA synthesis deficient mutant *opr3*. The application of exogenous MeJA can partially amend such difference in a time-dependent manner. Classification and pathway analysis of differential metabolites indicated that glucosinolates and tryptophan metabolism, amino acids and small peptides metabolism, lipid metabolism, especially fatty acyls metabolism were affected by endogenous JA deficiency and exogenous MeJA treatment.

Some isothiocyanates involved in plant resistance showed lower abundances in *opr3* than wild type, thus plant’s bacterial and insect resistance capability might be impaired by endogenous JA deficiency. No significant restorations were observed after MeJA treatment in *opr3*. But gene expression analysis revealed that the biosynthesis and hydrolysis of aliphatic glucosinolates related genes were down-regulated by endogenous JA deficiency and up-regulated by exogenous MeJA treatment. We suggest that plant resistance to bacteria and insects was impaired in the absence of endogenous JA and this impairment can be rescued by exogenous MeJA. Isothiocyanates that inhibit plant growth showed higher abundances in *opr3*, indicating plant growth may be inhibited in response to JA deficiency and the inhibition can be relieved by MeJA treatment. As a category of essential bioactive substances for plant defense, multiple glucosinolates and their hydrolysis products were influenced differentially by endogenous JA deficiency. This complex regulation may be due to diversity of their biological activities and their synthetic pathways.

The synthesis of serotonin was activated in *opr3*, indicating plants have other means of stress defense in the absence of endogenous JA. Similar trends of metabolites and genes involved in tryptophan-dependent auxin biosynthesis were observed, which indicated that this pathway was influenced by endogenous JA deficiency and exogenous MeJA treatment. α-linolenic acid metabolism was activated by MeJA treatments as evidenced by both metabolomics and gene expression analyses, supporting a positive feedback regulation of JA synthesis pathway. Many lipid metabolites showed variation patterns similar to that of α-linolenic acid, suggesting JA interacts with other lipids synergistically. We also found many small peptides regulated by JA, they may act as signaling molecules, further study is needed to elucidate their biological functions.

JAs are of great importance in plant growth, development and stress responses. Previous studies on JAs were principally focused on plant’s phenotypic and molecular aspects and lacked overall understanding of plant metabolites. In this study, we combined metabolomics and gene expression analyses to obtain comprehensive profiling of metabolites involved in JA functions and synthesis. The results provided a perspective view of the interaction and regulation network of plant metabolites regulated by JA, as well as a better understanding of interaction network of plant hormones and the mechanism of plant resistance.

## Methods

### Plant materials and MeJA treatment

*Arabidopsis thaliana* wild type Wassileskija (Ws) and mutant *opr3* were used in the experiment. The seeds were sown on 1/2 Murashige and Skoog medium after disinfection by 2% NaClO and 70% ethanol and vernalized at 4 °C for 3 days. The seedlings were transferred to pots after 10 days and cultivated in climate chamber (22 °C; 8/16 h light/dark cycle, 65% r. h.).

MeJA, Tween-20 (Sigma Aldrich) and deionized water (Millipore, Milford, MA, USA) were used to prepare 0.03% MeJA solution. The MeJA solution was sprayed on plant leaves after a vegetative growth period of approximately 28 days. The leaves were collected at different time points after treatment (0 h, 1 h, 4 h and 8 h), immediately frozen in liquid nitrogen and ground to fine powder. The powders were stored at −80 °C until analysis.

### Metabolite extraction

10 biological replicates were prepared for each genotype and treatment group. For each sample, 100 mg plant leaf powder was weighted and transferred to a 1.5 mL centrifuge tube. 750 μL water was added and the tube was placed in an ultrasonic cleaner (KunShan, China) for 30 min. After centrifugation (Centrifuge 5417 R, Eppendorf) at 15000 rpm for 10 min, the supernatant was transferred to another tube and 750 μL MeOH-ACN (1:1, v/v) was added, followed by sonication and centrifugation. The supernatants were combined and 400 μL of the mixture was dried using a vacuum concentrator (Concentrator plus, Eppendorf). The dried samples were redissolved in aliquots of 80 μL MeOH-H_2_O (1:1, v/v), filtered through a 0.1 μm membrane and transferred to sample vials for LC-MS analysis. Total protein was extracted by Phenol method and quantified by Bradford assay^59^. Three biological replicates were performed.

### UPLC-MS analysis

The plant extracts were analyzed using an UPLC-HRMS system (UPLC, ACQUITY UPLC H-Class Bio, Waters; MS, Q-Exactive, Thermo Scientific) equipped with a heated electrospray ionization (HESI) source. UPLC separation was performed on a BEH C18 column (2.1 × 100 mm, 1.7 μm, Waters) at a flow rate of 0.3 mL min^−1^. HPLC grade solvents and additives were from Fisher Scientific (ThermoFisher Scientific, NJ, USA). The gradient program using 0.1% FA in water (phase A) and 0.1% FA in ACN (phase B) was applied as follows: 95% A at 0 min to 55% A at 7 min, 5% A at 10 min and held for 4 min, then returned to initial condition. The column temperature was 35 °C and the injection volume was 5 μL.

MS analysis was performed in the positive ion mode. The instrument was calibrated using external standard before analysis to ensure mass accuracy of better than 3 ppm throughout the experiment. The HESI source parameters were as follows: Spray voltage at 3.5 kV, Capillary temperature at 320 °C, Sheath gas flow rate at 30 arb. units, Aux gas flow rate at 10 arb. units, Sweep gas flow rate at 5 arb. units, Heater temperature at 350 °C, S-lens RF level at 55. Full MS scan (*m/z* 70–1000) with resolution of 70 000 was used. 3 scans can be obtained per second, which provides sufficient data points for quantification. MS2 scan used normalized collision energy of 35 V, an isolation window of 0.8 *m/z* and a mass resolution of 35 000.

### Metabolomics data processing

Peak alignment, background subtraction and component extraction of the raw data were achieved by SIEVE 2.1 software (Thermo Scientific). Component extraction was performed between retention time 0.5 and 16 min with intensity threshold at 500 000, minimum scan at 9 and signal to noise ratio at 10. Principal component analysis (PCA), hierarchical cluster analysis (HCA), orthogonal partial least squares discriminant analysis (OPLS-DA) were performed using SIMCA-P 13 software (Umetrics) after data were scaled to Pareto variance. Statistical analyses of the quantitative data were performed using EXCEL and SAS. Compounds with CV < 20%, fold change >2 and p < 0.05 were picked for further identification.

Identification of differential compounds was achieved with the help of accurate mass measurement of molecular ions and fragment ions with high resolution. Accurate mass of molecular ions was searched against compound database (METLIN, KEGG, PlantCyc, AraCyc, HMDB, LIPID MAPS, MassBank, PubChem and MeSH) with mass accuracy at 5 ppm to generate a list of candidate chemical formulas. Isotopic pattern of the molecular ions helped to determine the probable formulas. MS/MS spectra database match was used to match the fragment ion spectra to the candidate compounds. MS/MS spectra were also compared with theoretical fragmentation pattern with mass accuracy at 10 ppm using Progenesis QI 2.0 (Waters). Metabolite identifications can be classified as level II based upon their spectral similarities with public/commercial spectral libraries in accordance with the MSI guidelines^35^.

Cluster analysis of identified differential compounds was achieved using Cluster 3.0 and TreeView. Pathway analysis was performed using MetaboAnalyst 3.0 (http://www.metaboanalyst.ca/MetaboAnalyst/faces/Secure/upload/PathUploadView.xhtml) and Pathway and Compound Databases (Plant Metabolic Netwok, MetaCyc, KEGG pathway) in TAIR (http://www.arabidopsis.org/portals/metabolome/metabolome database.jsp).

### Gene expression analysis

Total RNA was extracted using TRIzol reagent (Invitrogen, USA) according to manufacturer’s instructions. RNA concentration and purity were determined using a Nanodrop ND-1000 spectrophotometer (Nanodrop Technologies, Rockland, DE, USA), and RNA integrity was checked by 1% (w/v) agarose gel electrophoresis (data not shown). Genomic DNA elimination and first-strand cDNA synthesis were performed with a FastQuant RT Kit (Tiangen Biotech, Beijing, China) using 1.5 μg of total RNA. Three independent biological replicates for each sample were performed in this procedure.

RT-qPCR was performed using SYBR Premix Ex Taq (TaKaRa, Dalian, China) on a 7500 Fast Real-Time PCR System (Applied Biosystems, USA). Three technical replicates for each biological replicate were performed in the same qPCR run. The qPCR reaction system mix was prepared according to manufacturer’s instructions and subjected to an initial denaturation step of 95 °C/30 s, followed by 40 cycles of 95 °C/3 s, 60 °C/30 s. Finally, a melting curve was generated by increasing temperature from 65 to 95 °C in order to verify specificity of the amplification product. Gene-specific primers (a single peak in qPCR melting curve products) used were listed in Supplementary Table S4 and *ACTIN* was used as control. Relative expression values of all genes were calculated with the formula 2^−∆∆Ct^ using the cycle threshold (Ct) values and verified using One Way ANOVA (P < 0.05).

## Additional Information

**How to cite this article**: Cao, J. *et al*. Effects of MeJA on *Arabidopsis* metabolome under endogenous JA deficiency. *Sci. Rep.* **6**, 37674; doi: 10.1038/srep37674 (2016).

**Publisher's note:** Springer Nature remains neutral with regard to jurisdictional claims in published maps and institutional affiliations.

## Supplementary Material

Supplementary Tables and Figures

Supplementary Dataset 1
