# Supplementary material for: Effects of MeJA on Arabidopsis metabolome under endogenous JA deficiency
Source: Sci Rep. 2016 Nov 24;6:37674. doi: 10.1038/srep37674 (PMC5121592; doi:10.1038/srep37674)
Supplement: Supplementary Tables and Figures [file srep37674-s1.pdf]

Effects of MeJA on *Arabidopsis* metabolome under endogenous JA deficiency

Jingjing Cao, Mengya Li, Jian Chen, Pei Liu, Zhen Li

Supplementary Information

Table S1 Detailed information of extraction optimization

Extract	Solvent	Ratio (sample / solvent)	Condition	Concentration
A	Water, Organic	1:3	Sonication	None
B	Water, Organic	1:15	Sonication	None
C	Water, Organic	1:15	Sonication	Vacuum concentration
D	Water, Organic	1:15	Heating	Vacuum concentration

Table S2 List of identified compounds

	Name (level of identification)	Compound ID	RT (min)	m/z	Formula	m/z (Theor)	Mass error (ppm)	WT/*opr3* fold change (*p* value)	opr3_JA_1h/*opr3* fold change (*p* value)	opr3_JA_4h/*opr3* fold change (*p* value)	opr3_JA_8h/*opr3* fold change (*p* value)
1	Tetrahydrothiophene 1-oxide (II)	CSID1096	4.48	105.0371	C4H8OS	105.0369	2.26	0.15 (2.33E-11)	0.96 (3.46E-01)	0.32 (4.56E-15)	0.48 (1.96E-08)
2	4-Isothiocyanato-1-butene (II)	HMDB33867	5.47	114.0375	C5H7NS	114.0372	2.66	2.93 (3.79E-18)	1.15 (4.48E-03)	0.63 (5.04E-09)	0.92 (2.44E-01)
3	Proline (II)	HMDB00162	0.90	116.0710	C5H9NO2	116.0706	3.40	0.73 (2.67E-09)	1.15 (3.72E-03)	1.34 (2.24E-07)	0.46 (2.37E-15)
4	6-Acetyl-1,2,3,4-tetrahydropyridine (II)	HMDB30345	0.93	126.0916	C7H11NO	126.0913	2.06	0.40 (8.05E-10)	0.74 (3.43E-06)	0.83 (8.02E-03)	0.48 (1.32E-09)
5	(2E,4E)-2,4-Octadien-1-ol (II)	HMDB40151	5.40	127.1119	C8H14O	127.1117	1.25	0.75 (2.98E-03)	0.46 (4.60E-06)	0.80 (7.98E-03)	0.87 (6.48E-02)
6	5-Isothiocyanato-1-pentene (II)	HMDB34300	6.51	128.0530	C6H9NS	128.0528	1.20	3.25 (4.24E-15)	0.85 (2.03E-04)	0.52 (6.53E-14)	0.73 (3.42E-05)
7	p-Mentha-1,3,5,8-tetraene (II)	HMDB29641	8.02	133.1012	C10H12	133.1012	0.17	1.48 (1.78E-12)	2.73 (3.85E-08)	3.34 (2.48E-10)	3.09 (1.89E-11)
8	L-Glutamine (II)	HMDB00641	1.08	147.0764	C5H10N2O3	147.0764	0.13	0.47 (2.14E-08)	1.05 (2.05E-01)	0.75 (9.41E-06)	0.80 (2.71E-05)
9	5-Hydroxy-p-mentha-6,8-dien-2-one (II)	HMDB37011	11.06	149.0961	C10H14O2	149.0961	0.06	0.89 (2.51E-04)	0.88 (2.68E-03)	0.42 (3.01E-16)	0.31 (2.21E-18)
10	1-Methoxy-4-propylbenzene (II)	HMDB32626	8.06	151.1118	C10H14O	151.1117	0.39	1.40 (2.23E-10)	9.51 (3.07E-09)	11.52 (1.02E-10)	8.69 (1.84E-12)
11	2-Hydroxyadenine (II)	HMDB00403	4.84	152.0567	C5H5N5O	152.0567	0.09	0.42 (9.21E-11)	0.92 (1.00E-01)	0.54 (4.14E-12)	0.99 (8.66E-01)
12	3-Indoleacetonitrile (II)	HMDB06524	7.90	157.0760	C10H8N2	157.0760	0.16	0.38 (1.06E-12)	0.54 (7.17E-09)	2.78 (4.80E-07)	4.42 (8.38E-06)
13	Tranexamic Acid (II)	HMDB14447	0.92	158.1175	C8H15NO2	158.1176	0.35	0.38 (5.84E-10)	0.59 (7.11E-10)	0.78 (3.70E-04)	0.46 (5.37E-12)
14	Indoleacetaldehyde (II)	HMDB01190	6.15	160.0758	C10H9NO	160.0757	0.68	0.50 (1.07E-09)	0.74 (2.99E-06)	0.77 (2.75E-04)	0.68 (2.22E-07)
15	D-1,5-Anhydrofructose (II)	HMDB41561	0.89	163.0601	C6H10O5	163.0601	0.00	0.20 (7.58E-08)	0.63 (2.36E-06)	0.41 (2.84E-09)	0.32 (1.07E-10)
16	3-Methylsulfinylpropyl isothiocyanate (II)	HMDB06095	4.48	164.0197	C5H9NOS2	164.0198	0.80	0.09 (9.13E-12)	0.77 (2.85E-06)	0.23 (6.59E-17)	0.31 (4.48E-13)
17	(1beta,2beta,5beta)-p-Menth-3-ene-1,2,5-triol (II)	HMDB38978	11.06	169.1222	C10H18O3	169.1223	0.63	1.03 (2.35E-01)	0.91 (1.35E-02)	0.41 (1.12E-15)	0.30 (9.93E-18)
18	(E)-10-Hydroxy-8-decenoic acid (II)	HMDB39533	5.51	169.1223	C10H18O3	169.1223	0.04	1.34 (1.30E-11)	0.78 (6.13E-05)	1.68 (1.52E-08)	1.78 (2.24E-11)
19	2,4-Dimethylpimelic acid (II)	HMDB59760	11.06	171.1015	C9H16O4	171.1016	0.41	1.13 (3.89E-04)	0.96 (2.48E-01)	0.46 (7.92E-14)	0.35 (2.75E-16)
20	5-Hydroxyindoleacetaldehyde (II)	HMDB04073	6.77	176.0707	C10H9NO2	176.0706	0.54	0.45 (8.10E-08)	0.63 (1.99E-07)	0.82 (2.48E-04)	0.88 (1.30E-02)
21	2,5-Diisopropylphenol (II)	HMDB32579	11.33	179.1430	C12H18O	179.1430	0.23	6.63 (1.91E-16)	1.46 (1.71E-04)	5.08 (6.66E-10)	7.44 (2.22E-08)
22	6-(3-Hexenyl)tetrahydro-2H-pyran-2-one (II)	HMDB37829	6.11	183.1375	C11H18O2	183.1380	2.49	1.60 (1.23E-01)	1.45 (4.52E-02)	3.14 (4.65E-04)	1.42 (1.66E-01)
23	Glycyl-Isoleucine (II)	HMDB28844	2.89	189.1236	C8H16N2O3	189.1234	1.22	0.40 (2.86E-13)	0.82 (6.34E-04)	0.84 (4.35E-04)	1.00 (9.17E-01)
24	1-Isothiocyanato-5-(methylsulfinyl)pentane (II)	CSID178518	6.52	192.0511	C7H13NOS2	192.0511	0.17	2.24 (1.45E-20)	0.76 (4.04E-08)	0.45 (2.65E-16)	0.58 (1.52E-08)
25	Carbendazim (II)	HMDB31769	3.54	192.0775	C9H9N3O2	192.0768	3.89	0.38 (2.02E-12)	1.01 (8.19E-01)	1.10 (2.44E-02)	0.88 (1.59E-02)

26	Cucurbic acid (II)	HMDB29388	8.03	195.1375	C12H20O3	195.1380	2.52	0.49 (3.32E-14)	0.92 (6.51E-02)	0.43 (6.08E-14)	0.61 (5.67E-12)
27	Alanyl-Isoleucine (II)	HMDB28690	2.85	203.1391	C9H18N2O3	203.1390	0.40	0.30 (9.10E-15)	0.91 (4.79E-02)	0.82 (1.03E-04)	0.98 (7.19E-01)
28	4-Hydroxy-3-(3-methyl-2-butenyl) acetophenone (II)	HMDB30770	6.37	205.1220	C13H16O2	205.1223	1.49	1.10 (1.52E-03)	14.67 (9.30E-10)	10.97 (5.05E-09)	9.65 (8.53E-10)
29	Dhelwangin (II)	HMDB30703	7.18	207.1015	C12H16O4	207.1016	0.34	1.17 (7.67E-06)	2.13 (1.07E-07)	2.02 (1.06E-10)	1.89 (4.16E-13)
30	10beta-12,13-Dinor-8-oxo-6-eremophilen-11-al (II)	HMDB37604	9.57	207.1379	C13H18O2	207.1380	0.27	0.38 (1.46E-09)	0.71 (3.68E-08)	0.73 (6.71E-08)	0.76 (8.83E-07)
31	3,4-Methylenesebacic acid (II)	HMDB59729	4.80	209.1174	C12H18O4	209.1172	0.86	3.42 (1.60E-13)	28.01 (1.16E-10)	305.66 (1.75E-11)	551.57 (2.28E-13)
32	4,5-Dihydrovomifoliol (II)	HMDB40615	11.06	209.1535	C13H22O3	209.1536	0.51	1.00 (9.32E-01)	0.93 (7.38E-02)	0.38 (1.40E-16)	0.27 (7.85E-19)
33	Hydroxyphenylacetylglycine (II)	HMDB00735	1.92	210.0762	C10H11NO4	210.0761	0.55	0.45 (1.52E-07)	0.78 (3.18E-04)	0.86 (1.28E-02)	1.10 (4.11E-02)
34	Jasmonic acid (II)	HMDB32797	8.06	211.1327	C12H18O3	211.1329	0.81	1.20 (2.50E-04)	118.12 (1.13E-09)	133.93 (4.29E-17)	89.05 (1.36E-12)
35	2-Hydroxyacorenone (II)	HMDB30916	9.88	219.1741	C15H24O2	219.1743	1.10	2.46 (3.87E-15)	0.90 (3.08E-02)	2.33 (4.50E-07)	3.19 (3.37E-17)
36	Calamusenone (II)	HMDB38200	9.63	219.1743	C15H22O	219.1743	0.19	2.52 (1.19E-15)	0.88 (1.40E-02)	2.28 (1.44E-06)	3.06 (3.24E-16)
37	Glycyl-Phenylalanine (II)	HMDB28848	3.40	223.1077	C11H14N2O3	223.1077	0.08	0.33 (3.93E-10)	0.87 (8.01E-03)	0.82 (3.19E-04)	1.01 (7.36E-01)
38	(8alpha,10beta,11beta)-3-Hydroxy-4,15-dinor-1(5)-xanthen-12,8-olide (II)	HMDB37244	9.53	225.1482	C13H20O3	225.1485	1.43	3.96 (2.34E-09)	1.14 (2.78E-03)	2.58 (3.64E-07)	2.43 (6.67E-07)
39	Procurcumadiol (II)	HMDB34721	8.04	233.1534	C15H22O3	233.1536	0.89	2.88 (5.81E-21)	1.13 (2.47E-02)	2.34 (2.74E-14)	2.97 (2.62E-14)
40	Zedoarondiol (II)	HMDB36448	8.69	235.1692	C15H24O3	235.1693	0.24	1.82 (4.83E-16)	1.02 (6.12E-01)	1.59 (6.97E-09)	2.09 (2.11E-11)
41	Serinyl-Methionine (II)	HMDB29045	1.39	237.0904	C8H16N2O4S	237.0904	0.19	0.40 (2.03E-13)	0.80 (8.33E-05)	0.72 (8.03E-07)	0.85 (7.63E-05)
42	L-Pyridosine (II)	HMDB29443	3.50	237.1233	C12H18N2O4	237.1234	0.29	0.25 (1.68E-10)	1.01 (8.69E-01)	0.79 (1.37E-04)	1.04 (5.35E-01)
43	1,2,3,4-Tetramethoxy-5-(2-propenyl)benzene (II)	HMDB34046	6.53	239.1275	C13H18O4	239.1278	1.19	1.02 (5.57E-01)	14.61 (6.36E-10)	12.18 (1.46E-12)	8.24 (4.42E-11)
44	(2S)-2-(4-Nitrophenyl)succinic acid (II)	CSID643369	2.17	240.0499	C10H9NO6	240.0503	1.51	0.40 (2.59E-08)	0.96 (3.84E-01)	0.80 (1.01E-04)	1.08 (8.48E-02)
45	Demethoxyshogaol (II)	HMDB41250	9.63	247.1691	C16H22O2	247.1693	0.63	2.39 (3.26E-11)	0.99 (8.15E-01)	2.25 (2.00E-07)	2.40 (9.91E-09)
46	Ginsenoyne J (II)	HMDB40373	11.09	247.2057	C17H26O	247.2056	0.23	3.30 (2.03E-20)	1.08 (1.27E-01)	1.87 (4.16E-08)	1.90 (3.03E-10)
47	1-Isothiocyanato-9-(methylsulfinyl) nonane (II)	CSID8096856	9.90	248.1134	C11H21NOS2	248.1137	1.34	0.24 (5.60E-18)	0.79 (4.67E-06)	0.72 (4.01E-09)	0.75 (7.85E-08)
48	5-Hydroxyindoleacetylglycine (II)	HMDB04185	3.76	249.0864	C12H12N2O4	249.0870	2.34	0.29 (2.14E-14)	0.82 (4.09E-04)	0.76 (7.15E-06)	0.99 (8.84E-01)
49	Heliannuol C (II)	HMDB35131	8.63	249.1486	C15H20O3	249.1485	0.32	2.01 (1.09E-16)	0.90 (2.38E-02)	1.59 (1.44E-05)	2.01 (5.72E-09)
50	Dimethylbenzyl carbinyl hexanoate (II)	HMDB32228	10.23	249.1848	C16H24O2	249.1849	0.43	4.53 (5.71E-14)	1.02 (6.27E-01)	1.93 (1.03E-05)	1.91 (3.21E-10)
51	FS4 toxin (II)	HMDB37064	7.48	251.1638	C15H22O3	251.1642	1.48	3.09 (5.87E-20)	1.18 (1.77E-03)	2.41 (1.04E-11)	2.98 (4.19E-09)
52	3-Methyl-alpha-ionyl acetate (II)	HMDB37631	11.72	251.2005	C16H26O2	251.2006	0.23	1.06 (1.53E-01)	1.04 (3.53E-01)	0.76 (5.39E-08)	0.72 (2.65E-09)

53	Serinyl-Phenylalanine (II)	HMDB29046	3.26	253.1183	C12H16N2O4	253.1183	0.07	0.62 (1.34E-06)	1.01 (8.93E-01)	0.97 (6.01E-01)	1.12 (4.02E-02)
54	Methyl-3-(2,3-dihydroxy-3-methylbutyl)-4-hydroxybenzoate (II)	HMDB32796	5.74	255.1226	C13H18O5	255.1227	0.39	1.87 (5.00E-06)	175.08 (1.34E-09)	174.30 (6.67E-11)	169.41(1.10E-12)
55	1-(beta-D-Ribofuranosyl)-3- pyridiniumcarboxylate (II)	CSID141635	0.92	256.0815	C11H13NO6	256.0816	0.25	0.37 (5.75E-16)	0.91 (4.65E-03)	0.71 (1.75E-11)	0.71 (3.98E-11)
56	Nandrolone (II)	HMDB02725	10.14	257.1898	C18H26O2	257.1900	0.75	1.97 (3.36E-18)	0.97 (4.93E-01)	1.72 (3.78E-13)	2.08 (3.88E-13)
57	1-[(2,5-Dimethylphenyl)azo]-2-naphthalenol (II)	HMDB32889	8.71	259.1229	C18H16N2O	259.1230	0.29	0.23 (1.68E-07)	0.95 (5.44E-01)	0.50 (7.32E-06)	1.58 (4.03E-03)
58	Methionyl-Isoleucine (II)	HMDB28976	1.23	263.1423	C11H22N2O3S	263.1424	0.34	0.18 (1.25E-07)	0.66 (7.08E-05)	1.58 (2.37E-07)	1.53 (4.00E-07)
59	Phenylalanyl-Valine (II)	HMDB29008	3.82	265.1548	C14H20N2O3	265.1547	0.49	0.49 (8.38E-09)	0.89 (1.20E-01)	0.91 (9.23E-02)	1.09 (1.13E-01)
60	12-Oxo-2,3-dinor-10,15-phytodienoic acid (II)	HMDB32090	9.90	265.1797	C16H24O3	265.1798	0.46	4.55 (5.04E-12)	1.19 (6.94E-03)	4.56 (1.00E-07)	6.61 (7.97E-12)
61	4-Hydroxy-3-methoxy-2,10-bisaboladien-9-one (II)	HMDB33917	10.02	267.1952	C16H26O3	267.1955	1.01	4.63 (2.55E-11)	1.19 (4.60E-03)	4.00 (8.79E-08)	5.53 (3.32E-09)
62	Estradiol (II)	HMDB00151	9.03	273.1847	C18H24O2	273.1849	0.90	2.66 (2.60E-13)	0.95 (1.87E-01)	2.24 (2.34E-09)	2.71 (1.95E-09)
63	BR-Xanthone B (II)	HMDB33326	5.31	275.0549	C14H10O6	275.0550	0.42	1.65 (4.27E-13)	1.03 (5.54E-01)	1.30 (8.14E-06)	1.69 (1.73E-12)
64	19-Nor-5-androstenediol (II)	HMDB04590	10.97	277.2159	C18H28O2	277.2162	1.11	2.52 (1.58E-15)	1.00 (9.73E-01)	1.45 (1.78E-05)	1.49 (3.01E-07)
65	Alpha-linolenic acid (II)	HMDB01388	11.20	279.2316	C18H30O2	279.2319	0.92	3.55 (8.59E-21)	1.10 (7.14E-02)	1.97 (3.39E-06)	2.01 (3.01E-10)
66	Artemether (II)	HMDB15643	7.42	281.1743	C16H26O5	281.1747	1.55	2.64 (7.75E-20)	1.03 (6.00E-01)	3.07 (1.23E-08)	3.75 (3.98E-11)
67	Lactapiperanol C (II)	HMDB33630	9.23	283.1901	C16H26O4	283.1904	1.01	2.14 (6.20E-14)	0.89 (5.31E-02)	2.06 (7.56E-07)	2.26 (1.24E-09)
68	Tetraphyllin B (II)	HMDB29914	6.91	288.1083	C12H17NO7	288.1078	1.81	5.82 (1.40E-12)	1.19 (3.19E-03)	2.82 (4.15E-06)	3.96 (1.38E-09)
69	Estriol (II)	HMDB00153	9.85	289.1794	C18H24O3	289.1798	1.46	3.45 (2.65E-11)	1.10 (7.55E-02)	1.80 (1.85E-05)	2.31 (4.23E-08)
70	(9Z,11E,13E,15Z)-4-Oxo-9,11,13,15-octadecatetraenoic acid (II)	HMDB31098	9.02	291.1951	C18H26O3	291.1955	1.27	2.89 (6.43E-16)	1.01 (8.26E-01)	2.48 (5.99E-10)	2.91 (6.47E-10)
71	(2'E,4'Z,7'Z,8E)-Colnelenic acid (II)	HMDB30996	10.66	293.2105	C18H28O3	293.2111	2.12	4.23 (6.53E-18)	1.12 (1.73E-02)	3.37 (1.37E-08)	4.17 (9.28E-13)
72	Tyrosyl-Isoleucine (II)	HMDB29108	3.74	295.1653	C15H22N2O4	295.1652	0.22	0.49 (1.46E-09)	0.84 (3.11E-02)	0.83 (2.78E-03)	1.01 (7.99E-01)
73	13(S)-HOT (II)		10.20	295.2267	C18H30O3	295.2268	0.24	2.48 (3.97E-10)	0.99 (8.31E-01)	2.19 (7.26E-08)	2.45 (1.88E-08)
74	15-KETE (II)	HMDB10210	12.02	301.2158	C20H30O3	301.2162	1.35	2.06 (9.62E-14)	1.04 (3.66E-01)	2.15 (3.15E-10)	2.59 (2.37E-08)
75	Ascorbigen (II)	HMDB29839	5.24	306.0968	C15H15NO6	306.0972	1.35	12.05 (1.13E-11)	0.49 (2.94E-10)	6.65 (2.06E-05)	6.33 (1.48E-04)
76	5'-Carboxy-gamma-chromanol (II)	HMDB12799	9.85	307.1898	C18H26O4	307.1904	1.91	27.02 (1.12E-11)	1.96 (1.55E-07)	17.09 (1.14E-06)	22.05 (9.88E-08)
77	Corchorifatty acid A (II)	HMDB32664	9.72	309.2057	C18H28O4	309.2060	1.09	4.50 (1.37E-18)	1.03 (4.45E-01)	3.17 (2.18E-09)	3.48 (1.60E-09)
78	(9Z,11R,12S,13S,15Z)-12,13-Epoxy-11-hydroxy-	HMDB33505	10.03	311.2210	C18H30O4	311.2217	2.20	1.12 (3.38E-04)	0.75 (6.07E-07)	0.95 (1.90E-01)	0.86 (7.14E-04)

	9,15-octadecadienoic acid (II)										
79	Avocadene 1-acetate (II)	HMDB31043	11.20	311.2575	C19H36O4	311.2581	1.84	3.81 (2.28E-21)	0.87 (2.30E-02)	3.73 (6.81E-02)	4.16 (2.18E-16)
80	(±)-(E)-13-Hydroxy-10-oxo-11-octadecenoic acid (II)	HMDB40900	10.69	313.2368	C18H32O4	313.2373	1.71	1.68 (2.29E-10)	1.09 (7.51E-02)	1.31 (8.29E-03)	0.97 (5.93E-01)
81	Dehydrophytosphingosine (II)	HMDB38057	10.40	316.2842	C18H37NO3	316.2846	1.33	0.49 (5.90E-09)	0.68 (2.92E-06)	0.58 (9.49E-07)	0.58 (2.04E-06)
82	Phytocassane D (II)	HMDB41057	10.14	317.2105	C20H28O3	317.2111	1.96	1.86 (5.31E-16)	0.94 (1.63E-01)	1.74 (1.56E-13)	1.92 (3.27E-12)
83	Oryzalide B (II)	HMDB37592	7.99	321.2054	C19H28O4	321.2060	1.98	10.75 (4.12E-14)	1.67 (3.74E-07)	10.65 (2.23E-12)	13.82 (1.20E-11)
84	14,15-DiHETrE (II)	HMDB02265	11.33	321.2421	C20H34O4	321.2424	1.00	1.03 (5.46E-01)	1.10 (1.06E-01)	0.80 (3.17E-02)	0.60 (1.40E-05)
85	Alpha-Linolenoyl ethanolamide (II)	HMDB13624	11.50	322.2736	C20H35NO2	322.2741	1.41	0.46 (1.14E-06)	0.91 (1.48E-01)	0.60 (1.76E-06)	0.60 (1.94E-06)
86	2,3-Dinor-8-iso prostaglandin F2alpha (II)		8.81	327.2162	C18H30O5	327.2166	1.22	3.19 (8.82E-21)	1.10 (1.25E-01)	2.89 (1.27E-09)	3.28 (1.02E-11)
87	Tryptophyl-Phenylalanine (II)	HMDB29090	6.60	334.1548	C20H21N3O3	334.1550	0.61	0.39 (2.48E-11)	0.83 (1.28E-03)	0.48 (1.62E-10)	0.75 (1.46E-04)
88	9,10-DiHODE (II)	HMDB10221	10.77	335.2186	C18H32O4	335.2193	2.03	1.67 (2.89E-10)	0.74 (7.77E-07)	1.79 (2.08E-09)	1.76 (4.54E-09)
89	Cibaric acid (II)	HMDB38580	9.52	347.1825	C18H28O5	347.1829	1.14	1.81 (2.19E-11)	0.70 (5.95E-06)	1.61 (2.24E-06)	1.62 (2.34E-09)
90	Tetrahydrocortisol (II)	HMDB00949	8.54	349.2368	C21H34O5	349.2373	1.53	7.51 (1.43E-09)	2.25 (1.33E-07)	13.90 (5.80E-10)	26.49 (2.53E-10)
91	2,3-Dinor-8-iso prostaglandin F1alpha (II)		8.03	351.2134	C18H32O5	351.2142	2.26	0.23 (3.11E-13)	0.75 (4.47E-07)	0.24 (1.14E-18)	0.25 (8.73E-14)
92	Phe Pro Thr (II)		3.93	364.1865	C18H25N3O5	364.1867	0.54	0.49 (5.28E-13)	0.58 (8.94E-10)	0.65 (4.87E-10)	0.74 (2.71E-06)
93	D-1-[(3-Carboxypropyl)amino]-1-deoxyfructose (II)	HMDB38663	7.93	266.1235	C10H19NO7	266.1234	0.12	234.68 (6.00E-08)	62.81 (1.51E-02)	70.73 (1.50E-06)	58.41 (4.60E-04)
94	Cortolone (II)	HMDB03128	9.15	367.2466	C21H34O5	367.2479	3.54	3.72 (1.41E-11)	1.85 (4.90E-07)	5.08 (1.99E-07)	5.69 (3.83E-09)
95	Asp Lys Ile (II)		1.58	375.2234	C16H30N4O6	375.2238	1.10	1.24 (7.64E-06)	0.44 (2.18E-10)	0.97 (5.31E-01)	1.01 (9.12E-01)
96	Sarcostin (II)		8.19	383.2425	C21H34O6	383.2428	0.82	6.41 (8.82E-22)	1.45 (1.06E-04)	6.05 (1.74E-07)	8.12 (1.34E-08)
97	Prostaglandin E2 ethanolamide (II)	HMDB13038	7.52	396.2730	C22H37NO5	396.2744	3.66	4.40 (1.77E-05)	0.92 (7.42E-01)	3.20 (1.00E-04)	5.58 (1.93E-04)
98	PG(16:1(9Z)/0:0) (II)		11.13	465.2602	C22H43O9P	465.2612	2.11	5.66 (8.22E-14)	0.93 (2.99E-01)	4.34 (5.66E-10)	5.17 (3.50E-10)
99	1-Isothiocyanato-8-(methylsulfinyl)octane (II)		9.35	467.1884	C10H19ONS2	467.1889	1.04	0.38 (1.54E-09)	0.71 (3.80E-08)	0.73 (6.89E-08)	0.76 (7.86E-07)
100	Blumenol C O-[rhamnosyl-(1->6)-glucoside] (II)	HMDB31935	7.79	541.2609	C25H42O11	541.2619	1.91	2.68 (4.10E-16)	1.16 (1.52E-02)	2.44 (2.30E-15)	2.13 (6.66E-11)
101	3-Hydroxy-beta-ionol-3-[glucosyl-(1->6)-glucoside] (II)	HMDB37527	6.14	557.2561	C25H42O12	557.2568	1.34	3.18 (1.35E-13)	0.80 (5.60E-06)	2.08 (2.20E-08)	1.61 (1.91E-12)
102	Leukotriene F4 (II)	HMDB06465	9.12	569.2915	C28H44N2O8S	569.2891	4.19	1.85 (1.11E-10)	1.26 (8.03E-03)	2.58 (6.52E-08)	2.56 (6.82E-11)
103	Melilotoside A (II)	HMDB41464	11.32	613.4092	C35H58O7	613.4075	2.81	26.42 (5.48E-10)	1.54 (8.43E-05)	23.39 (8.45E-08)	46.81 (1.18E-08)
104	Sulforaphane (II)	HMDB05792	5.45	178.0353	C6H11NOS2	178.0355	1.25	1.90 (2.03E-18)	0.94 (7.68E-04)	0.43 (7.81E-16)	0.52 (2.26E-08)

105	1-Isothiocyanato-6-(methylsulfinyl) hexane (II)	HMDB38461	7.48	206.0668	C8H15NOS2	206.0668	0.00	1.23 (8.55E-09)	0.81 (1.47E-07)	0.70 (5.64E-11)	0.78 (1.80E-07)
106	1-Isothiocyanato-7-(methylsulfinyl) heptanes (II)	HMDB38441	8.47	220.0824	C9H17NOS2	220.0824	0.00	0.84 (1.63E-05)	0.80 (1.45E-07)	0.73 (1.69E-07)	0.71 (3.46E-10)
107	Glucobrassicanapin (II)	HMDB38417	4.37	388.0735	C12H21NO9S2	388.0730	1.16	4.04 (2.51E-03)	1.25 (4.29E-01)	47.90 (3.24E-11)	152.12 (5.71E-11)
108	Indolylmethyl-desulfoglucosinolate (II)		4.27	369.1113	C16H20N2O6S	369.1115	0.50	0.56 (7.09E-05)	1.29 (1.27E-03)	6.04 (9.23E-18)	25.78 (4.37E-10)
109	8-Methylthiooctyl glucosinolate (II)		6.60	478.1230	C16H31NO9S3	478.1234	0.77	0.00 (8.67E-06)	0.59 (4.00E-03)	0.15 (2.72E-05)	0.23 (5.50E-05)

Metabolite identifications can be classified as level II based upon their spectral similarities with public/commercial spectral libraries in accordance with the MSI guidelines

Table S3 Result from Pathway Analysis

	Total Cmpd	Hits	Raw p	-log(p)	Holm adjust	FDR	Impact
Arginine and proline metabolism	38	2	9.87E-18	39.157	9.87E-17	4.94E-17	0.09
Aminoacyl-tRNA biosynthesis	67	2	9.87E-18	39.157	9.87E-17	4.94E-17	0
alpha-linolenic acid metabolism	23	2	6.97E-16	34.899	5.58E-15	2.32E-15	0.16
Tryptophan metabolism	27	4	1.72E-15	33.995	1.21E-14	4.31E-15	0.29
Glucosinolate biosynthesis	54	1	5.41E-13	28.244	3.25E-12	1.08E-12	0.04
Biosynthesis of unsaturated fatty acids	42	1	7.84E-13	27.874	3.92E-12	1.31E-12	0
Alanine, aspartate and glutamate metabolism	22	1	1.33E-12	27.344	5.33E-12	1.33E-12	0.24
Purine metabolism	61	1	1.33E-12	27.344	5.33E-12	1.33E-12	0
Pyrimidine metabolism	38	1	1.33E-12	27.344	5.33E-12	1.33E-12	0
Nitrogen metabolism	15	1	1.33E-12	27.344	5.33E-12	1.33E-12	0

Table S4 Primer sequences for gene expression analysis by RT-qPCR

Gene Symbol	Accession No.	Description	Forward and reverse primer Sequence (5'-3')	Pathway
BCAT4	AT3G19710	Branched-chain aminotransferase 4	GCGAATGTCAAGTGGGAAGAG<br>TGGCCATACTGAAGAACAGCAG	Glucosinolates metabolism
MAM1	AT5G23010	Methylthioalkylmalate synthase 1	GCATTGTTCATCTCATGTTG<br>GCGGAGTGAGGGATCCACCA	
MAM3	AT5G23020	Methylthioalkymalate synthase-like	CTCTCTTCGCCTGACCCGTCCA<br>ACACGCACATAGTTCTTGTGG	
CYP79F1	AT1G16410	Cytochrome p450, family 79, subfamily f, polypeptide 1	CATGGAATGGACACTTGCGGA<br>TGAATGTGGCTACCTTTGGGA	
TGG1	AT5G26000	Myrosinase 1	GCAGCCAGTTACTACTACCCAAA<br>TTATCCCCAAGAGACCAAGC	
TGG2	AT5G25980	Myrosinase 2	GCTTCACTCACCGATACCCA<br>ACTCCAAGCTCTTCCATCACG	
UGT74B1	AT1G24100	UDP-glucosyl transferase 74b1	CACCACTACCTACACCGCCTCCTCA<br>GCTCAAAGACGGTAAGCCACGGATA	
SOT16	AT1G74100	Desulfo-glucosinolate sulfotransferase 16	CATGGGTTATGGATTCACTGAT<br>ATCTCAGGAGTCAAATAATTAGC	Tryptophan metabolism
CYP79B2	AT4G39950	Cytochrome p450, family 79, subfamily b, polypeptide 2	AAAGCTATCCTCCGCGAAGC<br>CATATCGGCTAAGAAGGACTTGAC	
CYP79B3	AT2G22330	Cytochrome p450, family 79, subfamily b, polypeptide 3	TCTACCGATGCTTACGGGATTG<br>TACAAGTTCCTTAATGGTTGGTTTG	
CYP71A13	AT2G30770	Cytochrome p450, family 71, subfamily a, polypeptide 13	ATTCGGATCAGGGAGAAGGATA<br>CGATACCAATGGCTTCAGTTAGAT	
NIT1	AT3G44310	Nitrilase 1	AAGCAAGGGAGCAGAGCTAGTGTT<br>TTTCCTAGCCACGTCAGCCAATCT	
NIT2	AT3G44300	Nitrilase 2	CTCATCCACCATTGTTCGAG<br>CTTGCAGCCTCCACAATAAA	
NIT3	AT3G44320	Nitrilase 3	ATGATCCTACTGTCTCCGGAGGTG<br>CCAAGATCAAGATCAGCTGTGACG	
AOS	AT5G42650	Allene oxide synthase	TGGTTATCGAAAGCCACGAC<br>AATCTCTCCGGCACAAACTC	α-linolenic acid metabolism
AOC1	AT3G25760	Allene oxide cyclase 1	CTCTCAGAACTTGGGAAATAC<br>GATCTCCGAGACCAAAACCTA	
AOC2	AT3G25770	Allene oxide cyclase 2	AATTAGATCGACACAGCCCCAAG<br>CCGAGACCGAACATTAAGCTGA	
AOC3	AT3G25780	Allene oxide cyclase 3	CGAAGGAGATAGAAACAGTCCAGC<br>CCGAGACAAAGCTCTGTTGGTT	
AOC4	AT1G13280	Allene oxide cyclase 4	AACGCATAGGAATCACCGCA<br>TGTGACCATAATCTCCGAAGTAGA	
OPR3	AT2G06050	12-Oxophytodienoate reductase 3	CATGCAGTGTATCAACCTAATG<br>CCAGCTCGAATCGCATTCAAAG	
JAZ10	AT5G13220	Jasmonate-zim-domain protein 10	CGAGTCGTCGATGGAGACAG<br>CTCGAGAAAACGTTGCAGTG	

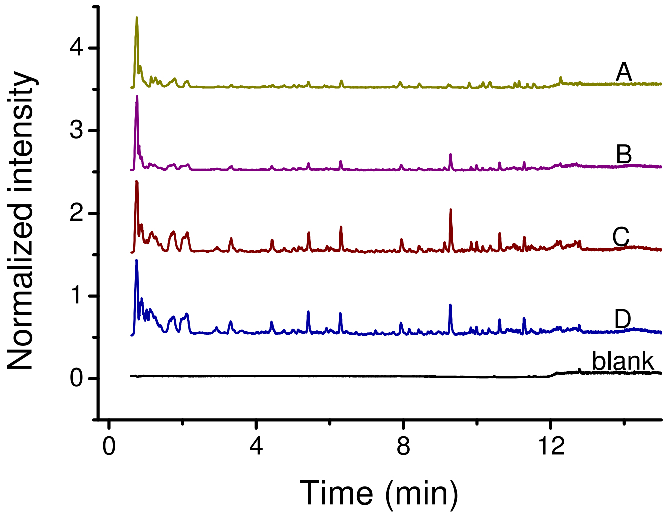

Fig. S1 UPLC-MS total ion chromatograms (TIC) of *Arabidopsis* leaves extracted by different methods

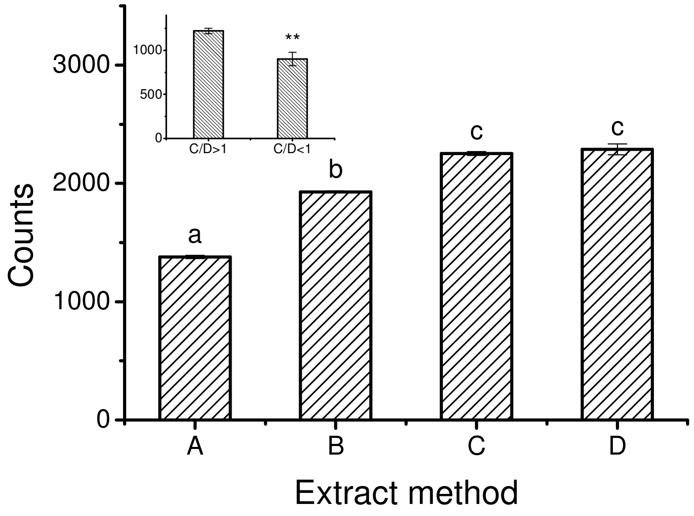

Fig. S2 Number of detected peaks (intensity threshold, 5×10^{6}) from different extract methods. Intensity comparison of detected peaks between method C and D (insert). Data represent mean ± SD from three replicates. Superscript letter and asterisk indicates the result of ANOVA test ($p<0.01$)

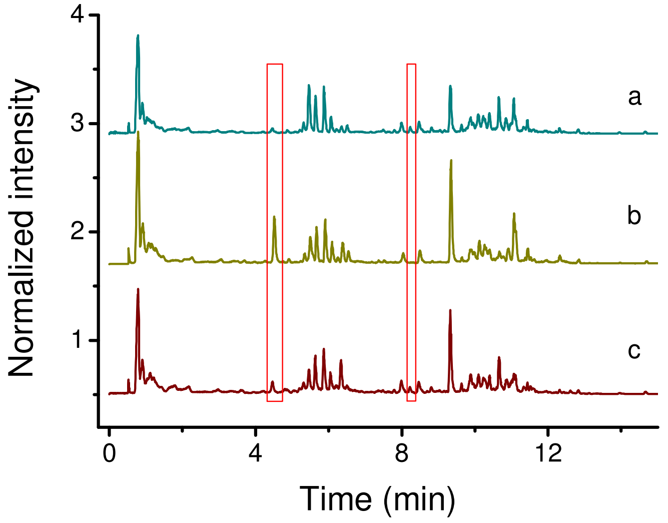

Fig. S3 TIC of *Arabidopsis* leaves extracts with different genotypes and treatments. **a** Wild type, **b** *opr3*, **c** *opr3* treated with MeJA for 8 h

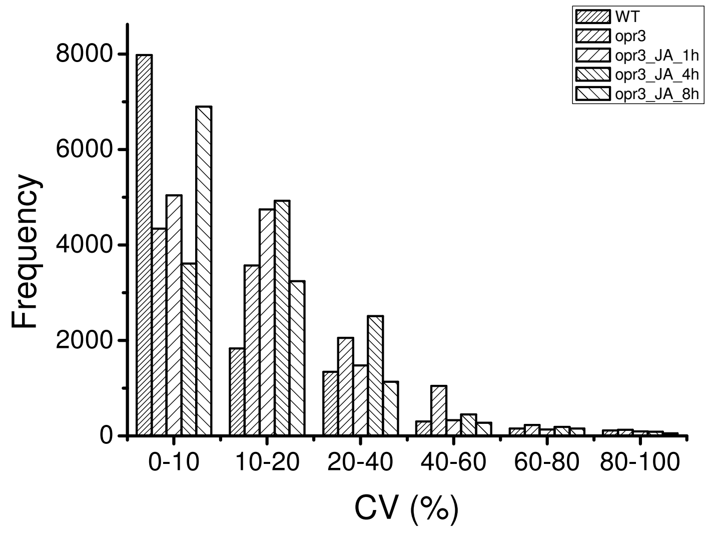

Fig. S4 Distribution of coefficient of variations of the 12016 signals

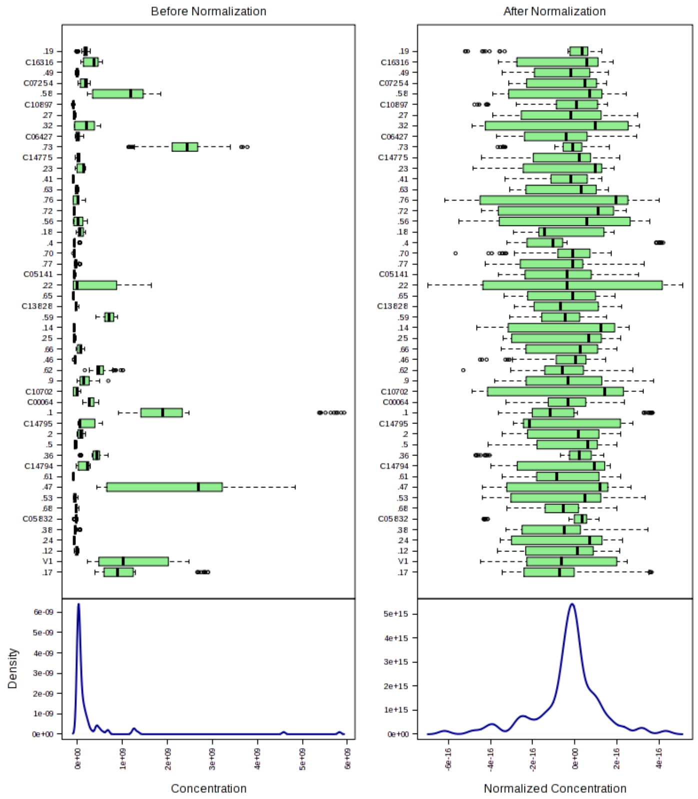

Fig. S5 Data normalization by sum, log transformation and Pareto scaling

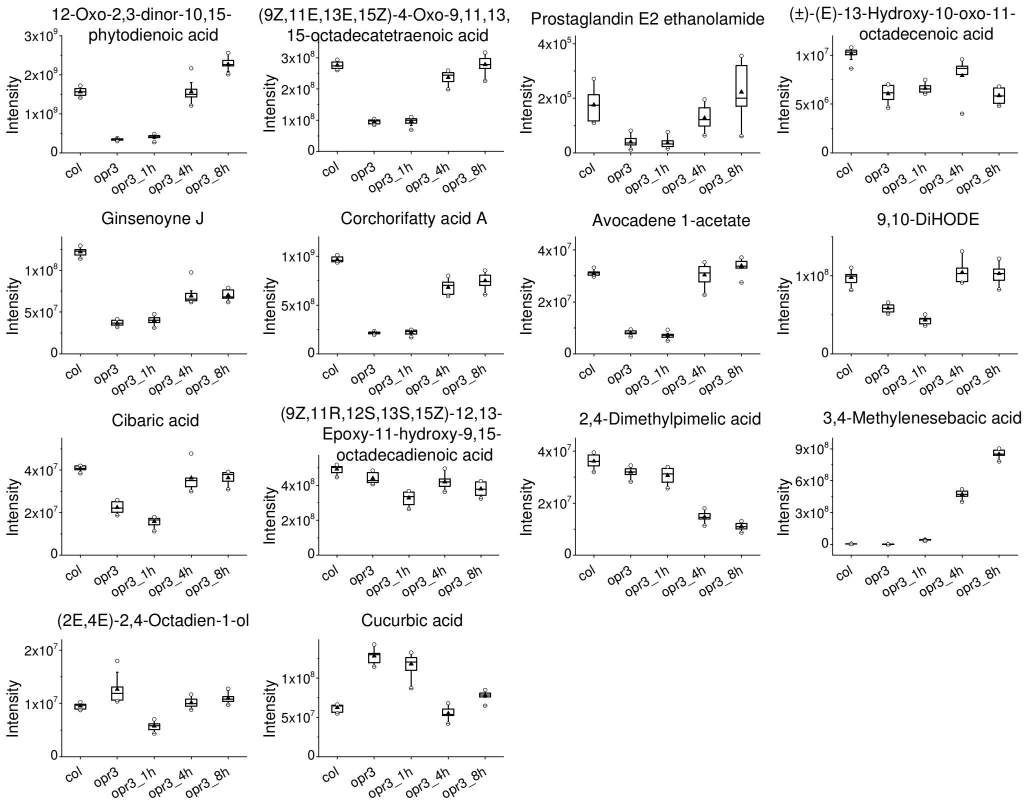

Fig. S6 Other fatty acyles impacted by *OPR3* deficiency and MeJA treatment

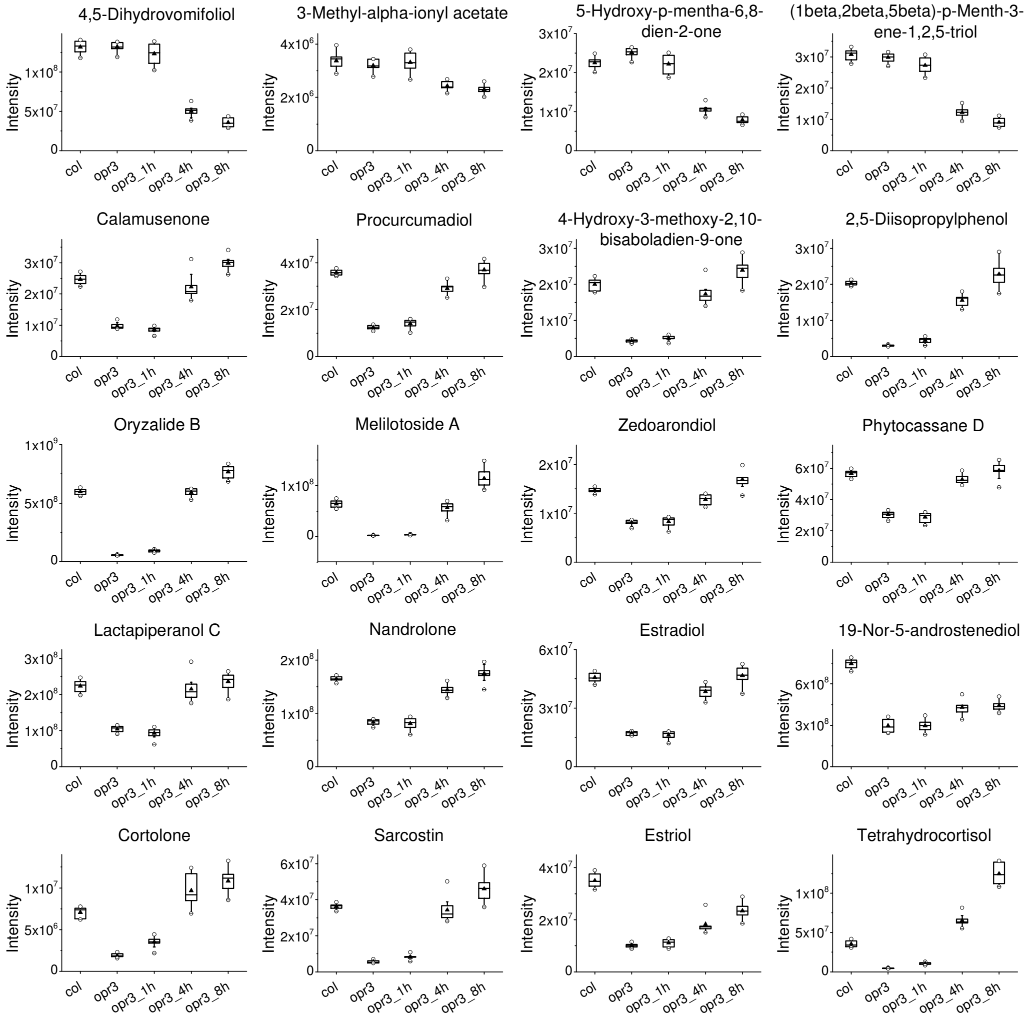

Fig. S7 Prenol lipids and sterol lipids were impacted by *OPR3* deficiency and MeJA treatment

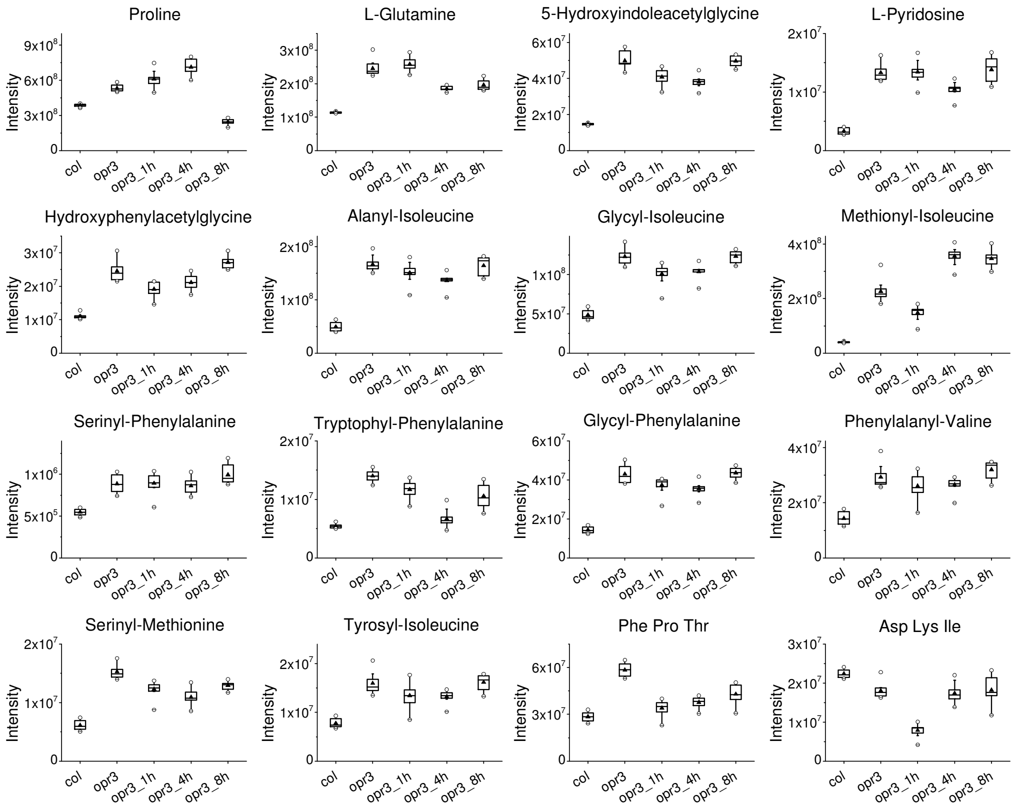

Fig. S8 Amino acid and derivatives, dipeptides and tripeptides were influenced by *OPR3* deficiency and MeJA treatment

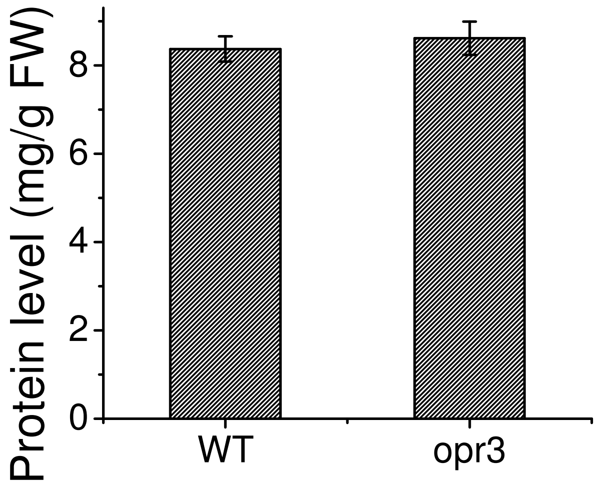

Fig. S9 Protein contents of wild type and *opr3* mutant by Bradford assay
